# Identifying Types of Eco-Anxiety, Eco-Guilt, Eco-Grief, and Eco-Coping in a Climate-Sensitive Population: A Qualitative Study

**DOI:** 10.3390/ijerph19042461

**Published:** 2022-02-21

**Authors:** Csilla Ágoston, Benedek Csaba, Bence Nagy, Zoltán Kőváry, Andrea Dúll, József Rácz, Zsolt Demetrovics

**Affiliations:** 1 Institute of People-Environment Transaction, ELTE Eötvös Loránd University, 1075 Budapest, Hungary; dull.andrea@ppk.elte.hu; 2 Doctoral School of Education, ELTE Eötvös Loránd University, 1075 Budapest, Hungary; nagy.bence@ppk.elte.hu; 3 Institute of Psychology, ELTE Eötvös Loránd University, 1064 Budapest, Hungary; csababenedek22@gmail.com (B.C.); kovary.zoltan@ppk.elte.hu (Z.K.); racz.jozsef@ppk.elte.hu (J.R.); zsolt.demetrovics@unigib.edu.gi (Z.D.); 4 Institute of Geography and Earth Sciences, ELTE Eötvös Loránd University, 1117 Budapest, Hungary; 5 Department of Sociology and Communication, Budapest University of Technology and Economics, 1111 Budapest, Hungary; 6 Centre of Excellence in Responsible Gaming, University of Gibraltar, Gibraltar GX11 1AA, UK

**Keywords:** eco-anxiety, eco-guilt, eco-grief, coping, pro-environmental behavior, climate change

## Abstract

Background: Climate change is one of the greatest challenges of the 21st century and it can affect mental health either directly through the experience of environmental traumas or indirectly through the experience of emotional distress and anxiety about the future. However, it is not clear what possible subtypes of the emerging “psychoterratic” syndromes such as eco-anxiety, eco-guilt, and eco-grief exist, how much distress they may cause, and to what extent they facilitate ecofriendly behavior. Methods: We analyzed semi-structured interviews (N = 17) focusing on the thoughts, emotions, and behaviors related to climate change by using a combination of inductive and deductive qualitative methods. Results and conclusions: The interviews revealed six eco-anxiety components, eight types of eco-guilt, and two types of eco-grief that help to understand the multifactorial nature of these phenomena. The six categories of coping strategies are in line with traditional coping models, and they are linked in various ways to pro-environmental behavior and the management of negative emotions. The results can help practitioners to gain a deeper understanding of emotions related to climate change and how to cope with them, and researchers to develop comprehensive measurement tools to assess these emotions.

## 1. Introduction

Besides the undeniable effects of climate change on hydrological and terrestrial systems [1], climate change also affects people’s physical and mental health [2,3]. People develop different emotions about climate change such as depression, anxiety, and anger, which affect behavior and well-being differently [4]. Emotional consequences of climate change can be extremely important to certain populations, such as indigenous peoples, whose common practices are disrupted by climatic and consequential environmental change [5], or activists who are greatly involved in collective action [6,7]. Albrecht [8] suggested that chronic stress on ecosystems is likely to produce chronic stress in humans, which results in certain “psychoterratic” or earth-related mental health syndromes, such as eco-anxiety, eco-paralysis, solastalgia, and eco-nostalgia. Eco-anxiety is a special type of stress and worry, which is related to the ecological crisis, and can be interpreted in the framework of existential and psychodynamic psychology as well as social sciences [9]. Worry about climate change is very common among children and young people (84% of them expressed at least moderate worry in a large-scale study) [10]. Eco-paralysis is characterized by the inability to meaningfully respond to the climatic and ecological challenges [8], and it can stem from either the sudden emotional shock caused by the threat or cognitive dilemma of having too many and sometimes conflicting options for action [11]. Another important phenomena is eco-guilt that occurs when people realize they have violated personal or social standards of behavior [12] and eco-grief, which is a response to ecological loss that can be related to the loss of physical environment, anticipated future losses and the disruptions to environmental knowledge systems, which leads to the feeling of loss of identity [13]. Solastalgia is a concept akin to eco-grief; it describes the anguish or despair we feel when we realize that the place we live in and love is chronically deteriorating, and the comfort—or solace—we derive from the current state of our home environment is gradually disappearing. Compared to solastalgia, where people have a “lived experience” of the change process, eco-nostalgia is experienced when people return to a location that has been entirely transformed in their absence due to development or climate change [8].

In the last couple of years, increasing interest has been directed towards eco-emotions. Pihkala [14] conducted a preliminary exploration of the taxonomy of climate emotions and created the following categories: surprise-related emotions (which included both positive emotions such as amazement and negative emotions such as shock and trauma), threat-related emotions (e.g., fear, worry), strong anxiety-related feelings, sadness-related emotions (such as sadness, grief, solastalgia), strong depression-related feelings, emotions closely related to guilt and shame, emotions related to indignation, disgust-related emotions, anger-related emotions, feelings of hostility (which included contempt, skepticism and feeling bored), envy-related emotions and several kinds of positive emotions (e.g., interest, empowerment, joy, pride, gratitude, etc.). In the current study, we focused on those phenomena that were the most frequently discussed in relevant studies according to Pihkala [14], namely fear/worry/anxiety, sadness/grief (which are related to the aforementioned “psychoterratic syndromes), guilt/shame, and—in relation to coping—hope/empowerment.

Although there is a growing body of research exploring the emotions associated with climate change, it is still not clear whether these negative emotional states or emerging mental health syndromes are necessarily maladaptive or pathological and whether they should be mitigated due to their potentially paralyzing effect on ecofriendly behavior [15] or, on the contrary, whether they have a positive influence on taking ecofriendly actions [16,17]. It is also possible that the various degrees and types of these emotional states as well as the different mindsets [18] related to them affect behavior in different ways (e.g., the lower amount of stress may increase the willingness to act). In a qualitative study with climate activists from Sweden, Denmark, and the Global South, Kleres and Wettergren [6] found that both hope and guilt can act as catalysts that transform potentially paralyzing fear into action-oriented anger, although in different ways. A recent quantitative study found that eco-anger predicted better mental health outcomes than eco-anxiety and eco-depression, and both eco-anger and eco-depression predicted greater engagement in collective action [4]. According to some previous studies, evoking guilt could lead to actual pro-environmental behavior [19] or at least behavioral intentions [12,20], although these intentions do not necessarily result in actual behavior [21]. Collective guilt may be a better predictor of the willingness to engage in mitigation behaviors than eco-anxiety [22]. On the other hand, eco-guilt may lead to ecofriendly behavior only if reparation suggestions are presented in an appropriate way [23]. There may be cross-cultural differences between emotions that are more likely to motivate pro-environmental behavior. Caillaud, Krauth-Gruber, and Bonnot [24] found that empathy for victims is more likely to motivate action among German respondents, while guilt and sadness are more likely to motivate action among French respondents.

Another important question is whether the traditional coping responses, namely problem-focused and emotion-focused coping [25], could also be fully applied to coping with climate change and the negative emotions that come with it and whether these coping responses are considered to be adaptive or maladaptive regarding climate change mitigation and the person’s well-being. Previous studies considered certain strategies, such as having a problem-solving attitude or seeking social support to be more adaptive, and others, like avoidance, denial, unrealistic optimism, or wishful thinking, to be rather maladaptive, at least from a climate change mitigation perspective [26]. On the other hand, problem-focused strategies can have some unfavorable consequences: when stressors are less controllable—like in the case of climate change—relying solely on problem-focused strategies could lead to more distress [27]. Meaning-focused coping—which was introduced as a new category of coping by Folkman [28]—is an appraisal-based coping strategy in which the person relies on their beliefs, values and existential goals to sustain coping as well as well-being during the times of chronic stress; this strategy can be a more advantageous one to apply when we face the challenges of climate change [27].

Although several previously mentioned theoretical papers draw attention to the possible mental health consequences of climate change [2,3,8,9,15] or the prevalence of different emotions related to climate change [29,30,31], only a few studies have examined the concrete and tangible manifestations of them and the possible subtypes of these phenomena [13]. Moreover, the majority of empirical studies that focus on “psychoterratic syndromes” or—more broadly—eco-emotions are quantitative [14], so we know less about the specific qualities and possible subcategories of these phenomena. Therefore, the first aim of this paper was to provide an in-depth qualitative analysis of climate change-related psychological phenomena, such as eco-anxiety, eco-guilt, and eco-grief, in a climate-sensitive population in Central Europe and thereby help to define these concepts more precisely for future research and assist practitioners working with climate-sensitive populations. Another aim of our study was to further examine the possible ways of “eco-coping” and reveal which of the coping strategies can be considered as adaptive responses that also reinforce pro-environmental behavior. 

## 2. Materials and Methods

### 2.1. Procedure and Measures

We used semi-structured interviews to explore people’s experiences regarding climate change. The questions (Appendix A) aimed to reveal associations, attitudes, emotions, behavioral intentions, and concrete behaviors related to climate change, the perceived effects of climate change, and the sources of getting information about the topic. The study was approved by the Research Ethics Committee of the Faculty of Education and Psychology, ethics approval No.: 2019/379.

Before the interviews, the interviewers completed multistage training: first they acquired the theoretical basics, then they conducted pilot interviews. They then discussed their experiences with the research supervisors and finalized the interview protocol before the actual interviews were carried out. The interviewees were not previously acquainted with the interviewers and did not receive any incentives for participation in the interviews.

The participants signed an informed consent form prior to data collection in which they agreed to participate in the study. The interviews were audio-recorded, then anonymized, and verbatim transcripts were prepared by the research assistants. The length of the interviews ranged from 20 to 72 min. Because the topic of the interview could evoke inconvenient feelings, we also shared the contact information of a helpline with the participants.

### 2.2. Participants

The sample (N = 17) was recruited through social media advertisements and word of mouth in January 2020 by targeting a population that was thought to be more climate-sensitive due to their profession, strong interest in the topic, or experience of the impact of climate change. In the recruitment process, we did not apply strict criteria for climate sensitivity but considered the subjective experience of potential participants, i.e., participants could volunteer based on whether they felt affected. We chose this approach because some of the direct climate hazards, like wildfires, hurricanes, and sea level rise, which would increase vulnerability, are practically nonexistent in Hungary, while others, such as heatwaves, affect everyone as Hungary is a small country. The participants explained their exposure to climate change or their involvement in the issue during the interviews (e.g., higher sensitivity to changes in barometric pressure). Some of the interviewees were approached based on the recommendations of other interviewees based on whether they perceived them to be climate-sensitive. This sample size was sufficient to establish better understanding of these phenomena and small enough to leave room for more in-depth analysis [32]. The sample included environmental activists, participants with professions related to environmental issues or sustainable development (including students who studied related disciplines), as well as participants who were interested in the topic in general and were committed to climate change mitigation or felt affected by climate change; 6 male (35%) and 11 female interviewees participated in the study, and the mean age was 31.1 years (SD = 14.8) (Table 1).

### 2.3. Analysis

The interviews were analyzed using the combination of inductive and deductive qualitative approaches. As we wanted to contribute to the conceptualization and in-depth analysis of eco-anxiety, eco-paralysis, eco-guilt, eco-grief, and eco-coping, we chose to use the coding process of thematic analysis (TA), which is a method for systematically identifying and organizing patterns of meanings across a dataset and which enabled us to explore the complexity and reveal the deeper meaning of these phenomena [33]. For the initial coding we decided to choose a predominantly inductive, experiential, and rather semantic orientation [33]. This method allowed us to formulate a relevant theory—or, in this case, a deeper understanding and a possible data-driven categorization—based on the systematic analysis of the data rather than test preestablished hypotheses. 

For the multistep coding process of the transcript of the interviews (total length: 71,186 words) and for forming memos, we used Atlas.ti Cloud^TM^. The first step of the analysis was initial coding. In this phase, we did not use pre-defined categories and coded every extract of data that was potentially relevant [33]. After familiarizing with the dataset, the interviews were analyzed sentence-by-sentence and divided into content units. The highlighted information or a meaningful content unit could be a sentence or even a single word. A portion of data could be coded with more than one code [33]. The initial descriptive coding of the interviews was performed by the first and second authors who discussed and compared the interviews and formed memos based on the discussions. Initial coding also served as the primary data reduction. A total of 3217 codes were generated during this step of the analysis.

Based on the analysis of the memos and the discussions and through reexamining and analyzing the interview sections marked with the initial descriptive codes, we generated themes, which included those initial codes that shared some unifying features [33]. The themes represent “some level of patterned response or meaning within the data set” [34] (p. 82). The following themes were identified: Emotions, Sense of being affected by climate change, Key experiences, Relationship with nature, Past, Present, Future, Fantasy, Responsibility, Participant’s own action, Other’s action, Age/generational differences, Characterization of people/groups, Conflicts, Social influence, Hypothetical/desired/necessary actions, Specific actions implemented, International comparison, Explanations, Processes, Specific phenomena, Gathering information, Skepticism, Ambivalence/irony, Metaphors, Quitting from the context of the interview. The themes are listed and illustrated with examples in Appendix B.

It is important to note that an initial code could belong to several themes. For example, code “angry because of the news” was added to themes “Gathering information” and “Emotions”.

In the next phase, the developed themes were reviewed by the first and third authors. In this step, we incorporated inductive and deductive methods. We used the concepts of “psychoterratic” syndromes and coping mechanisms defined in the literature [8,13,23,25,26,35,36] for reorganizing the themes developed during the second step of the analysis in order to provide a coherent description of these constructs. Since we followed an inductive approach and the generation of codes was not the ultimate goal, but rather part of the development of the themes, we did not use quantitative measures to assess the intercoder agreement [37]. The coders worked independently but consulted regularly with each other at different stages of coding and compared the selected excerpts at the final stage. For those excerpts that were selected by only one of the coders, the coders discussed the reasons of the discrepancy until they reached consensus on whether to keep or discard the particular excerpt. This way we detected 99 excerpts related to eco-anxiety, 21 excerpts related to eco-guilt, 18 excerpts related to eco-grief, and 60 excerpts related to coping mechanisms.

## 3. Results

### 3.1. Eco-Anxiety

Six distinct components of eco-anxiety emerged from the interviews (examples for each category can be found in Table 2). The first category was the worry because of the Future and next generations which included worry for one’s own descendants and for the next generation in general. The second category was called Empathy. The quotes in this category reflected some kind of “secondary suffering”: the participants experience negative emotions because they see others (e.g., vulnerable populations, animals) suffer. The third category was Conflicts with family, friends, or colleagues derived from the different attitudes or behavior regarding climate change mitigation. These conflicts were accompanied by negative emotions in the interviews, usually anger or frustration. The fourth category comprised Being disturbed by the changes of the environment (e.g., droughts, warmer summers, no snow in winters, disappearance of plants or animals) that result in physical symptoms (e.g., swelling of body parts, panic attack-like symptoms associated with heatwaves, increased occurrence of allergic reactions) or lead to confusion or uncertainty. The fifth category incorporated Mental health symptoms, which are in line with the symptoms of anxiety disorders (e.g., generalized anxiety disorder, panic disorder) and mood disorders (e.g., depression) according to DSM-5 [38]. The sixth category was Helplessness and frustration caused partly by the magnitude of the challenge and partly by the lack of control over it.

### 3.2. Eco-Guilt

We identified eight types of eco-guilt (examples for each category can be found in Table 3). Two of them—Prophetic individual responsibility and Self-criticism, self-examination, self-blame—are considered to be more adaptive as they were accompanied by the mentioning of ecofriendly behavior and the sense of purpose. Prophetic individual responsibility included the participants’ sudden recognition of humanity’s or their own environmental impact, which was often described as an overwhelming burden. We called it prophetic because the participants reported that they had realized they knew more about the topic than other people, which made them feel responsible for enlightening others or evoked a desire to make others aware of the damage or impending disaster because otherwise there would be no change. The category of Self-criticism, self-examination, self-blame often reflected a self-improving trend: the participants reviewed how they could live an even more ecofriendly life and modified their behavior accordingly in several cases. However, self-torturing and self-exposing statements were also recurring elements in this category: the interviewees often talked about forgetting to do something or not doing something they could otherwise do.

Guilt/individual responsibility criticism was an interesting and somewhat ambivalent category: the participants who provided such statements did not consider guilt to be adaptive as it encourages action that does not necessarily help climate change mitigation. They shifted the responsibility to companies and thought that individuals should be more radical and apply more pressure on companies.

In comparison, Dissatisfaction with one’s actions and Feeling guilty about one’s past may be less adaptive as they mostly contain only criticism and dwelling on negative emotions with fewer behavioral attempts. Sometimes the participants devalued their past or present actions or felt that their actions had only a slight impact on the global processes. The topic of passivity often appeared here as well; they felt guilty about watching the process from the outside and not actually participating in it.

System maintenance guilt, Dilemma of harm and Guilt for one’s existence are also types of guilt that are less likely to redound to better mental health and pro-environmental behavior. The participants who reported System maintenance guilt felt that they were part of a system that is bad, but they could not control or quit it nonetheless; this is somewhat similar to learned helplessness [39]. They blamed a larger system (capitalism, globalization) for climate change but at the same time felt remorseful that they are parts, servers, and beneficiaries of this system and thus also contribute to the destruction of the environment. The Dilemma of harm was the best indicator of eco-paralysis [8]: the participants, due to the abundance of new information, were unable to decide whether their actions were good for the environment or not. For some interviewees, this led to the abandonment of any actions, but for others, it was a short-lived condition. Guilt for one’s existence is considered to be a more severe version of the Dilemma of harm: here, the participants felt that their mere existence was harmful and pointless.

This section may be divided by subheadings. It should provide a concise and precise description of the experimental results, their interpretation, as well as the experimental conclusions that can be drawn.

### 3.3. Eco-Grief

We managed to identify two types of eco-grief: the Loss of the physical environment and species and Anticipated future losses (Table 4). The first category included the losses that had already occurred. The participants often talked about losses that did not belong to their immediate environment (e.g., extinction of tropical animal species, melting of glaciers), and only some of the losses were related to their lives more directly (e.g., the loss of snowy winters). A sense of irreversibility often appeared in this category, frequently accompanied by anger. The second category, Anticipated future losses, included losses that had not happened yet, but the participants anticipated them in the near or moderately distant future. This kind of losses was often accompanied by sadness and often contained a human-centered perspective (namely, the participants regretted things disappearing because they would not be able to see them).

### 3.4. Coping Mechanisms

We identified six types of coping mechanisms for coping with eco-anxiety, eco-guilt, and eco-grief (examples for each category can be found in Table 5). Taking actions/planning—when the participants tried to adopt more ecofriendly actions in their lives or planned to do so in the near future—and Confrontation—when the participants tried to persuade other people to behave more ecofriendly, even at the cost of having conflicts with them—were categorized as problem-focused coping. The participants who accepted that they could only help locally, in their own environment, often found further worry unnecessary. They felt that they did what they could or needed to and sometimes encouraged the interviewers as well to take action in their own environment. A recurring theme was the joy and gratitude they felt for the small steps and small changes. Although both strategies seemed to be accompanied by more ecofriendly actions, they might affect the participants’ emotional states differently: while Taking actions was often accompanied by a decrease in negative emotions based on the interviews, Confrontation tended to intensify them in many cases.

On the other hand, three emotion-focused categories emerged from the interviews: Positive appraisal/optimism, Withdrawal/acceptance, and Problem avoidance/denial/wishful thinking. Positive reappraisal/optimism included the participant’s attempt to reframe the threat of climate change and interpret it as a challenge and focus on the positive and adaptive things that people could do—this incorporated the belief in the goodness of humanity and God and creative new inventions. While this more optimistic mindset could potentially reduce anxiety and stimulate taking action, at the same time it may include the danger that the person starts to think of climate change mitigation with an external locus of control [40], waiting for an automatic solution (this is reflected in the answer of Participant 13 and in the second quote of Participant 16 in Table 5). Withdrawal/acceptance reflects a somewhat cynical attitude, again reminiscent of the phenomenon of learned helplessness [39]. While in the case of System maintenance guilt the helplessness resulted from the participant’s feeling of being unable to quit a bad system, in the case of Withdrawal/acceptance, the participants feel helpless because they think it is too late to act and therefore conclude that the best solution is to accept the inevitable destiny of the humanity. Problem avoidance/denial/wishful thinking served to mitigate the participants’ negative emotions by averting their focus from the topic of climate change. This incorporated simply avoiding climate change-related news, focusing on more pleasant hobbies (e.g., sports, arts), or having pleasant fantasies about what people should do to make the world a better place. By using this strategy, the participants might have decreased their guilt and anxiety in a less adaptive way because their focus could also be averted from ecofriendly behavior.

The last category was seeking Social support, which is a mixed strategy as it contains both problem-focused and emotion-focused elements. The participants seeking social support often mentioned joining a community where environmental protection was an important topic. Thus, in these communities, they not only felt a sense of belonging and received emotional support, but also helped each other to develop pro-environmental behavior, shared new tips, and took on the role of environmental activists. Emotional support was experienced in different ways by the participants who mentioned social coping: in these environments (i.e., self-organizing eco-groups, activist groups), they had the opportunity to vent their pent-up negative emotions, but it was also important for them to be able to meet like-minded people and even form new friendships while getting away from a milieu insensitive to the problem. Social coping was the closest to meaning-focused coping: the participants who engaged in this kind of coping could draw on their pro-environmental beliefs and values and were able to experience hope because—together with their peers—they could do something for a greater purpose. This hope is able to fuel both coping with the chronic stress of climate change and maintain their well-being.

**Figure 1:**
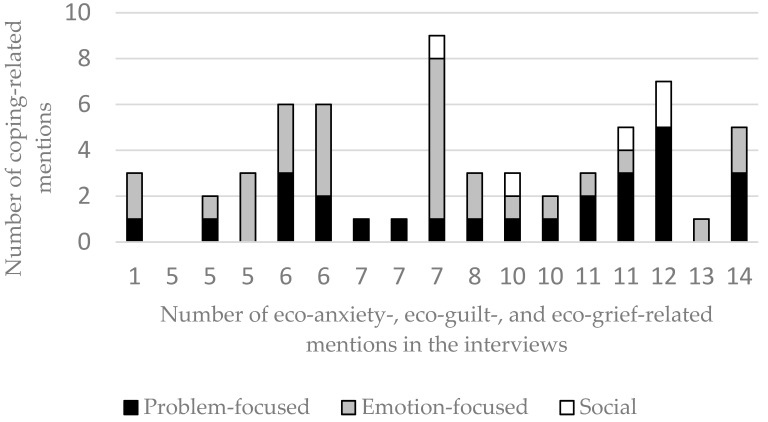
The relationship between the number of mentions of eco-anxiety, eco-grief, and eco-guilt and the various ways of coping in each interview. Note: The horizontal axis shows the data for the seventeen participants, in ascending order based on the number of “psychoterratic” symptoms, which is shown below each bar. The different colors (black, grey, white) of the bars indicate the different types of coping mechanisms. The vertical axis shows the number of the coping mechanisms mentioned by the participants.

In the final step, we quantified and summed up the quotes that were related to eco-anxiety, eco-guilt, and eco-grief, and we did the same for the coping strategies (separately for the problem-focused, emotion-focused, and social support-related quotes) in order to gain a better understanding of the pattern of coping strategies as a function of the number of “psychoterratic” symptoms. As Figure 1 shows, there is no clear relationship between the types of coping strategies and the number of symptoms.

## 4. Discussion

The results of our exploratory study indicate that eco-anxiety, eco-guilt, and eco-grief are multifactorial constructs, and future large-scale quantitative studies should consider their possible subtypes since they could impact ecofriendly behavior differently. The results suggest that some of the general symptoms of anxiety disorders can be applied to eco-anxiety as well, and they may cause temporary functional impairment. However, the results also showed that most of the participants successfully mobilized various coping strategies to alleviate anxiety, and some of these strategies proved to be adaptive, including from an ecological perspective, in addition to alleviating the symptoms of eco-anxiety. Our results concerning the themes in eco-anxiety are partially in line with the results of the current qualitative studies: helplessness, worry for future generations, as well as symptoms of anxiety have been found in other studies as well, while taking notice of changes in the weather was less prominent, and conflicts with others did not appear directly in other studies [41].

The eco-guilt categories presented in the results are partly consistent with those in previous studies. For example, the operationalization of guilt in a previous study [36] is somewhat similar to System maintenance guilt, which is some kind of collective guilt, while some examples that resemble Self-criticism, self-examination, self-blame appeared in the report of Coyle and van Susteren [42]. As we mentioned before, the Dilemma of harm is the equivalent of eco-paralysis [8], and this draws attention to the importance of sending clear messages to the public that set a clear direction for pro-environmental behavior. However, the appearance of Guilt/individual responsibility criticism warns that people do not react unanimously to arousing guilt, and it can cause the opposite effect. The fact that some of the participants felt Guilt for their mere existence suggests that eco-guilt and eco-anxiety can be interpreted as an existential phenomenon that emerges from our “ecological unconscious” as a reaction to the destruction of the natural environment [43]. Considering that guilt could be rooted so deeply, instead of just trying to get rid of it like any other illness, it would be preferable to gain a better understanding of it and, as a result of the understanding, allow it to bring quality development into our lives [43]. The types of eco-grief we identified were in line with two of the three categories described by Cunsolo and Ellis [13], but no further content categories emerged. This may highlight the intercultural differences in grieving and the fact that certain types of grief (e.g., the grief associated with disruptions to environmental knowledge systems and the resulting feelings of loss of identity identified by Cunsolo and Ellis [13]) may apply more to those who not only live closer to nature, but also experience the effects of climate change more intensely due to their geographical location (e.g., the Inuit in Labrador and family farmers from the Australian Wheatbelt).

Overall, the types of coping mechanisms that emerged in the interviews were partly in line with the general types of coping mechanisms [25,28,44] and the coping mechanisms related to climate change [26,45]. Certain themes we found in our study were related to the two main themes that were revealed in a scoping review of interventions for individual and group treatment of eco-anxiety [46]: problem-focused coping is related to encouraging clients to take action while social coping is similar to helping clients find social connection and emotional support by joining groups. Our results indicate that, in the right circumstances, climate-sensitive individuals instinctively discover those coping methods that professionals find effective as well. Based on the interviews, problem-focused coping and meaning-focused coping (which manifested in social coping in the narratives) indeed seemed to be more adaptive in general. When we consider adaptivity, we refer to Bradley et al.’s [47] definition of psychological adaptation, which is understood as a set of cognitive, affective, and behavioral responses to the challenge of climate change that involves acknowledging the problem, shifting attitudes to a more pro-environmental position, and adopting a problem-solving attitude, which contributes to psychological adjustment and emotion management. Namely, an adaptive response serves both the cause of climate change mitigation and individual well-being. Interestingly, although several participants were satisfied with their everyday actions, others reported dissatisfaction and devaluated the same actions. Based on the interviews, it seems that those who were thinking more locally could better accept their own limits and were less dissatisfied than those who had a more global perspective. It is worth examining in future studies whether certain personality traits (e.g., perfectionism) contribute to dissatisfaction with local action. The results suggest that overly threatening information and increased feelings of helplessness may lead to maladaptive coping strategies (e.g., problem avoidance/denial). The lack of a clear relationship between the number of quotes related to negative emotions/symptoms and coping patterns may be due to the fact that some of the participants who mentioned fewer negative emotions but more coping strategies experienced these negative emotions in the past (e.g., Participant 10).

### Limitations

It is important to note that guilt may partly appear as a byproduct of the interview process itself. According to Mallett [36], we feel eco-guilt if we are reminded of the fact that we or our group harm the environment or if we are reminded that we do not meet certain norms set by our society or ourselves. Presumably, the question related to responsibility served as a reminder of these norms. This may also explain why self-blaming statements appeared and why some interviews nearly turned into eco-guilt confessions [48].

Another possible limitation of this study is the small and selective sample. We strongly focused on individuals who were climate change-sensitive in some ways; therefore, our conclusions apply mainly to this group. The sample size was quite small; therefore, it is possible that the saturation of each theme may not have been fully achieved. The sample was selective, based on subjective feelings of vulnerability, so it may be necessary to target samples that are affected by the direct impacts of climate change (e.g., natural disasters), so that presumably more of the lived emotional experiences would be related to the theme of eco-grief, which—unlike eco-anxiety and eco-guilt—has received fewer mentions in the current research. It would also be interesting to examine in future research how these phenomena appear in less affected groups and how they are related to behavioral intentions.

## 5. Conclusions

To sum up, in order to understand and describe the psychological effects of climate change and deal with the suffering and difficulties associated with it, it is necessary to examine what people actually experience. The present study is one in a line of research conducted around the world that aims to achieve this goal. The next stage in this process is to develop a comprehensive measurement tool for assessing eco-anxiety, eco-guilt, and eco-grief based on interviews and the literature that can capture the complex nature of “psychoterratic” syndromes. This objective dovetails well with the trends in questionnaire development on eco-anxiety [17,49] and climate change worry [50] that ran in parallel with this research. The results can also help the work of mental health professionals who work with clients who experience eco-anxiety, eco-grief, or eco-guilt [46].

## Figures and Tables

**Table 1:** The main characteristics of the sample.

Participant ID	Sex	Age	Residence	Relevant Expertise/Profession
1	Male	28	Capital city	Chemical engineer, Master’s degree in Sustainable Development
2	Male	28	Capital city	Committed to climate change mitigation
3	Male	19	Another city	Has a strong opinion on the topic
4	Male	20	Another city	Psychology student
5	Male	30	Village	Animal rights activist, animal caretaker
6	Male	22	Another city	Student in teacher training with environment-related subject specialization
7	Female	20	Capital city	Committed to climate change mitigation, Sociology student
8	Female	77	Capital city	Strong climate sensitivity due to age, previously worked as a statistician
9	Female	20	Capital city	Environmental activist (Extinction Rebellion)
10	Female	23	Village	Conservation engineer
11	Female	35	Capital city	Committed to climate change mitigation, yoga instructor
12	Female	30	Capital city	Committed to climate change mitigation
13	Female	22	Capital city	Interested in climate change mitigation, but not yet an activist
14	Female	52	Another city	Teacher (English and Chemistry)
15	Female	26	Another city	Environmental activist, environmental Facebook group administrator, social worker
16	Female	43	Capital city	Committed to climate change mitigation, teacher, very religious (Christian)
17	Female	33	Capital city	Environmental activist (Extinction Rebellion)

**Table 2:** Aspects of eco-anxiety.

Category	Example
**Future and next generations**	“But if we don’t start taking some drastic steps for this matter, future generations will come off badly. Anyway, I feel really sorry for the next generation, I’ve already been feeling sorry for my future children for what they will be born into if people don’t bring about some huge change” (Participant 6).
**Empathy**	“Yeah, so a lot of people can already feel its direct effects. I’m a social worker, I’ve worked in elderly care, and I see how much the number of deaths is accelerating in the winter or summer period” (Participant 15). “For example, there was this documentary on Netflix, and David Attenborough’s series, and although it didn’t aim to blame people, but it pointed out that the way we live [is harmful/unsustainable], and the animals are those who feel the direct effects of it, and they are not able to do anything about it; and only we, people, can do something about it” (Participant 15).
**Conflicts**	“Well, I haven’t experienced this [eco-anxiety] so continuously, so strongly, but I always feel that I’m worried about it, but less strongly. But there were two occasions when I felt it very strongly. Once, after reading this study, and then I cried and told my mom that everything is meaningless and things like that. And yet I think I’m basically a pretty optimistic person. The other time was when we travelled to Gran Canaria for the second time this summer. I mean, we were there last year and the flight there was a total of 11 h, I mean, it was 11 h back and forth. And then I saw that the people there were all so cheery and it was like there was nothing wrong and I became so upset that I kept talking about it and that made my mom and brother angry, and they told me to shut up. And ironically, a few days after we had left a huge forest area caught fire, and the forest burned down also in places that we had visited earlier. So that was very tough” (Participant 9).
**Being disturbed by the changes**	“And really, there were times when we worried about when it was going to rain. Though people can really worry about a lot of things in their lives, this has not been among them so far. This has been a given so far, we haven’t had to worry about nature because nature has been doing its duties. But we meddled with it so much that the whole thing has become completely unpredictable, and this unpredictability is not good for anyone” (Participant 17).
**Mental health symptoms**	“There are times when I feel anger. Or panic. Yes, I definitely feel panic sometimes. For example, when I think about how many clothes are burned every year in the fast fashion industry. I mean, I don’t live my life like I’m cooking in the kitchen and I panic about it, but when I think about all of it that oh my God, what is this all about, how much oil flows into the Danube or the sea or anywhere… So, yes, I panic” (Participant 15).
**Helplessness, frustration**	“I used to see one article a month, and then when you read every day that the planet is in flames, we’re going to die, everything is bad, no one is helping anyone, I think it induces a process: I’m dealing with this topic more and because I feel helpless, that’s why I’m anxious” (Participant 12).

**Table 3:** Aspects of eco-guilt.

Category	Example
**Prophetic individual responsibility**	“And if I don’t acquaint people with the idea, explain it to them, educate them, and show the harm, then they won’t understand and then they won’t do anything” (Participant 5).
**Self-criticism, self-examination, self-blame**	“(…) Or, for example, when I thought about how many sanitary pads I was going to use as a woman, a fertile woman, in my following 20–30 active years. And I decided to switch to reusable sanitary pads on my 27th birthday” (Participant 15). “I ask myself from time to time what else I could do to act in a more environmentally friendly manner” (Participant 2).
**Guilt/individual responsibility criticism**	“I had a very interesting conversation with one of my acquaintances. He was the one who said that he thinks this whole environmentalism is harmful to the environment. In fact, the individual contribution to pollution is so little compared to how much waste factories emit. And by persuading the well-educated, wealthier classes who could lobby factories to facilitate change… With all sorts of bullshit messages like “Recycle!”, “Turn off the lights!”, “Be vegan!”, and so on… they feel like they’re doing something for the environment. But it rather prevents them from going above a certain level of anxiety and tension so they don’t force factories to make radical changes” (Participant 13).
**Dissatisfaction with one’s actions**	“I often feel that what I can do is far from enough” (Participant 7). “I also feel a little bit guilty that I don’t really care about this topic right now because there are some very enthusiastic climate advocates even in the closest circle of my friends. And when I somehow scrambled home between two parties yesterday, I had a kebab in my hand, and I was so full that I threw away the leftover. And I’ve been feeling guilty ever since” (Participant 4).
**Feeling guilty about one’s past**	“(…) I also felt something like that, that the way I have lived over the last 10–30 years has also contributed to this” (Participant 17). “Then there is the issue of housekeeping, for example. I was kind of a compulsive cleaner, a person who kept everything clean. I couldn’t stand it if there was a stain on the table or counter and… I used very, very strong chemicals” (Participant 15).
**System maintenance guilt**	“(…) Because of the daily comfort of people and the whole system—capitalism and globalism—built on it. And because of them, it is simply inevitable that you will be a part of it” (Participant 17).
**Dilemma of harm**	“At the same time, I have to think about it, if my products include tropical fruit in January, which is not seasonal at all, am I doing more damage than he does by using plastic bags?” (Participant 15).
**Guilt for one’s existence**	“(…) But then I felt such remorse that I produce a lot of unnecessary rubbish just through my existence, and it really caused unpleasant feelings on a daily basis. And then I tortured myself a lot by saying that the way I exist and… I don’t know, my needs and the things I do, like my job, are not indispensable… and it was so depressing” (Participant 16).

**Table 4:** Examples for eco-grief.

Category	Example
**Loss of the physical environment and species**	“It also makes me sad because I have noticed that I don’t see a lot of plants anymore that I used to see when I was young. I have never really dealt with insects, I mean like in more detail, but I have read about them dying en masse. But what I terribly miss is birds. We’ve been travelling to Őrség [a region in Hungary] every year for 10 years, and when we first went there in the summer, I remember what we saw there, what kind of bird chatter greeted us. And now I look for them in vain” (Participant 8).
**Anticipated future losses**	“Well, on the one hand, I grew up watching the movies of David Attenborough and I like the diversity of species very much, and when I read about extinction and decreasing populations, it breaks my heart that this diversity and beauty is withering. And the things I could see in the nineties, and in the current documentaries of David Attenborough… it is possible that in two years we could see only in video recordings how birds-of-paradise flutter in the forest, and it sucks” (Participant 1).

**Table 5:** Coping mechanisms related to eco-anxiety, eco-guilt, and eco-grief.

Category	Example
**Taking actions/planning**	“And to be honest, after I joined Extinction Rebellion, I felt for the first time that I was doing something useful or I was actually doing something, and maybe that helped, too” (Participant 9). “(…) To give back to nature what belongs to nature. To be involved more actively in producing even less garbage. You know, this zero waste thing, which I haven’t achieved yet… it is very difficult to achieve anyway. So, I have tips like that. I think that from the moment a person is actively involved in action, the whole situation becomes less threatening…” (Participant 6).
**Confrontation**	“And then that’s when I get upset. Really, I can give an example from my narrowest circle of friends that I do have to tell them not to ask for a straw for their drink, it should be so obvious, why would you need it? You can drink your beverage without it, why is it so important now?” (Participant 6). “What really bothered me was people’s indifference. I didn’t know what to do with that. Their bad decisions… I had such a defining moment last year that although I liked those people, I was really angry with them because they took the car to a dining place that was only 5 min from us just because it was hot outside. And I looked at them like ‘Are you serious?’” (Participant 7).
**Positive reappraisal, optimism**	“So, I don’t think there will be such a big cataclysm here, I still believe in human goodness and eagerness for action” (Participant 10). “I wouldn’t really say I’m pessimistic and depressed about it, but let’s say it’s urging and motivating me” (Participant 16). “I can see that a lot of things are not worth worrying about. Technological innovation is the thing that definitely needs to be done, because it is so great, and it’s so good to read about it, and it’s really a matter of power games” (Participant 13). “These consequences may be known to some extent, so I’m not really worried about it [climate change], and I also know that God holds all of this in His hands, and I know what the end of this will be because the end will be a happy ending” (Participant 16).
**Withdrawal/acceptance**	“(…) The phrase ‘environmental protection’ is truly misleading because it is much more about the protection of humanity. Because the environment will survive and regenerate anyway, but people will not” (Participant 13). “Well, it’s such a very weird thing, because there are these five stages of grief, and these can also be applied to those who are diagnosed with a terminal illness. And then denial is the very first, you know. Then, when you get over it, you accept it in some way. And these five stages appeared alternately during the summer. Because along with Greta Thunberg, it all intensified, more and more people started sharing the news about it. And the UPFSI or UPFRS, I don’t know the acronym right now, has a channel on YouTube and I’ve seen two videos there, which have already made it clear that this is irreversible, and a lot of lectures, too. And Jem Bendel who worked for the IPCC or made reports, if I’m not wrong, and I think he’s working as a climate change researcher at the University of Bristol, he has already started a grief therapy group” (Participant 11).
**Problem avoidance/denial/** **wishful thinking**	“But since I haven’t opened these articles anymore lately so they wouldn’t haunt me, I can only say what I thought about this half a year ago. Since then, I don’t know how bad the situation has become, but I don’t think it has changed because I haven’t heard that some miracle has happened and that everyone has really reacted to it” (Participant 12). “Well, what I can do to block this out is that I try to achieve different flow experiences by learning, doing yoga, fine arts, or some, I don’t know, cultural programs” (Participant 11). “But I’ve been thinking about it, and it would be so nice if people suspended all their actions for five years, didn’t go to college, work, but rather cleaned up the Earth, got the garbage out of the oceans, and figured out what to do with the garbage” (Participant 7).
**Social support**	“And then the way I was able to take part in Extinction Rebellion, it [the eco-anxiety] eased and I don’t think about it every day anymore, but only sometimes when I see the news, I get sad about it. This community can give me a lot and it’s very motivating that I’m in a company where others think and feel the same way I do. And the good thing about this company is that we do not talk about this topic all the time but we do talk about it as well, and that dissolves it all, and at the same time a community is forming that I’m very happy to join” (Participant 9). “It really bothered me that nobody in my milieu cared about these things. And then I secretly started looking for communities that cared, reading, and attending talks and things like that, where this climate issue is more in the focus of attention” (Participant 17).

## Data Availability

Anonymized transcripts of the interviews (in Hungarian) are available on request from the corresponding author.

## Appendix A

**Table A1:** The questions of the interview.

What is your first association with climate change?
What do you think about the credibility of the news about climate change?
Where do you find information on environmental/climate change topics? How do you select these resources?
How does climate change currently affect your life?
How do you think climate change will affect the lives of future generations?
How do you feel when you think about climate change?
Can you recall anything that you think has changed permanently and irreversibly as a result of climate change? Please tell us more about this.
Who do you think is responsible for protecting the environment and mitigating climate change? Why?
What do you think people do in general to protect the environment or stop climate change?
What do you do to protect the environment?
What do you think other people would do (and what they would not do) to protect the environment or stop climate change?
All in all, what are the most important environmental issues that you think we need to address at the societal level or at the individual level?

## Appendix B

**Table A2:** Description of the themes.

Theme Names	Description of the Themes	Example of the Initial Code in the Theme
Emotions	Emotions explicitly or indirectly expressed in the interview, quotes with a strong emotional charge	Was disturbed by the sight of too cheerful people
Sense of being affected by climate change	Personal episode or event related to the effects of climate change experienced by the participants on their own	Increased symptoms related to barometric pressure
Key experiences	An event or a concrete experience that can be considered as a turning point in the participants’ attitudes in terms of climate change	A book made her aware of the gravity of the situation
Relationship with nature	Codes that describe the participants’ relation to nature	Respect for each other’s living space
Past	Codes concerning the past (e.g., what the weather was like in the past, what was different, or what the participant did in the past)	Bees, birds, plants—everything has become different
Present	Codes concerning the present	There are many more forest fires these days
Future	Visions, expected events, happenings, scenarios related to climate change	More and more things will have to be imported to Hungary
Fantasy	The participants’ desires, dreams, wishful thinking	Conducting solar energy from space to the Earth by using a huge particle accelerator
Responsibility	The participants’ statements regarding responsibility	The responsibility of multinational companies is far greater than that of individuals
Participant’s own action	Steps, actions, participation taken by the participant	Uses fewer chemicals in the household
Other’s action	Codes describing the actions, non-actions, and behavior of others mentioned by the participant	Others may be willing to collect waste separately
Age/generational differences	Codes for generational differences, statements about childhood or the elderly	It is more difficult for older people to develop new habits
Characterization of people/groups	Statements about specific individuals (e.g., Greta Thunberg) or groups (e.g., climate change deniers, politicians, people who live in cities, etc.) and general descriptions of human nature, which can be considered as a kind of folk psychological characterization	Grew up in the countryside where love of nature and living things is a fundamental value for people
Conflicts	Quotes that reflect conflicting situations resulting from the participants’ sustainable behavior	Conflict with the family because she talks too much about the topic
Social influence	Codes related to persuading people, changing their behavior, the possible means of influence, the participants’ influence on other people	The influence of celebrities on policymakers
Hypothetical/desired/necessary actions	What further steps the participants consider important, what they hope for, what would be needed; quotes related to the planned or desired personal actions	Instead of individual action, pressure should be put on factories
Specific actions implemented	Statements about measures taken, organizations, inventions	Several zero packaging stores opened in Budapest
International comparison	Comparison of countries, evaluation of climate change measures of other countries or their own country (Hungary)	In developed societies, there are more movements that draw people’s attention to environmental protection
Explanations	The explanations the participants give about certain phenomena related to climate change, the theories they have, as well as the factors and moral issues that hinder sustainable behavior	The question of livelihood always overrides our attention to the environment
Processes	The participants present various interacting phenomena and processes	Deforestation disrupted an island’s ecosystem
Specific phenomena	Specific events and phenomena observed in nature; polluting activities	True bugs do not freeze in winter
Gathering information	Codes for gathering news and information related to climate change	The authenticity of news is difficult to verify
Skepticism	Doubts about climate change mitigation actions, leaders and decisionmakers, and climate change itself	There have been forest fires before
Ambivalence/irony	Codes related to self-mocking humor; the participants’ self-reflected responses and ambivalence about some actions	Questioning one’s own credibility on the subject
Metaphors	The participants characterize a phenomenon related to climate change with a metaphor or a poetic description	As if we were sitting on a sinking ship
Quitting from the context of the interview	The participants reflect on the interview situation or address the interviewer (e.g., they want to convince the interviewer of something)	Advises the interviewer to visit a website
